# Clinical outcomes after emergency transarterial renal embolization: a retrospective study

**DOI:** 10.1186/s42155-024-00505-y

**Published:** 2024-12-18

**Authors:** Rémi Grange, Nicolas Magand, Noémie Lutz, Julien Lanoiselee, Stéphanie Leroy, Claire Boutet, Sylvain Grange

**Affiliations:** 1 Department of Radiology, University Hospital of Saint-Etienne, Avenue Albert Raimond, Saint-Priest-en-Jarez, 42270 France; 2 Department of Anesthesia, University Hospital of Saint-Etienne, Saint-Priest-en-Jarez, France

**Keywords:** Kidney, Trauma, Transarterial embolization, Bleeding, Emergency

## Abstract

**Background:**

Studies on emergency transarterial embolization (TAE) of renal arterial injuries are rare. The aim of this retrospective study was to evaluate clinical outcomes after emergency transarterial renal embolization.

**Material and methods:**

Between January 1st, 2013 and January 1st, 2024, all consecutive patients treated for renal arterial injuries by TAE in emergency settings were retrospectively reviewed. Demographic, biological and angiographic data were recorded. The inclusion criteria were all patients ≥ 18-years-old treated by emergency TAE for renal vascular injury. Clinical success was defined as the resolution of bleeding signs without the need for repeat TAE, surgery, death related to massive blood loss during this period, without functional impairment (> 50% of parenchyma volume or onset of chronic kidney disease) following TAE.

**Results:**

During the inclusion period, 79 procedures were performed. The median age was 60[39–73] years old. On preoperative CT, ≥ 1 pseudoaneurysm was detected in 36(45.6%) patients, and active bleeding in 47(65.8%) patients. The preoperative median haemoglobin rate was 8.9[7.6–11] g/dl, and 37(46.8%) patients required red blood cell transfusions. The main aetiologies of arterial injury were blunt trauma (*n* = 19) and renal biopsy (*n* = 17). No severe adverse events were reported. Clinical success was reported in 74(93.7%) of the procedures. Three (3.8%) repeat embolizations were required, and were clinically successful. During the median follow-up of 7[1.5–35.5] months, 9(11.4%) patients died, of which 5(6.3%) occurred within 30 days.

**Conclusion:**

The present study reports high clinical success, low complications and low rebleeding rates of emergency renal TAE.

## Background

The mechanism of renal arterial injury includes blunt trauma, iatrogenic injuries, tumoral bleeding, or arteriovenous malformations [1]. Iatrogenic injuries can be secondary to renal artery angioplasties, JJ stents, percutaneous biopsies or partial nephrectomies [2, 3]. The number of iatrogenic arterial injuries has increased in recent years due to more systematic conduct of renal biopsies for kidney disease, tumour diagnosis and percutaneous nephrostomies [4–6]. Furthermore, the development of minimally invasive surgery has led to an increase in the rate of partial nephrectomies compared to radical nephrectomies [7]. The rate of significant bleeding after partial nephrectomies is estimated to be 3–5% [8, 9]. Renal injuries following blunt traumas, typically not life-threatening lower-grade injuries, can be categorized into renal lacerations, renal contusions, and renal vascular injuries. Additionally, spontaneous bleeding of angiomyolipoma, cyst, or clear-cell carcinoma are classic causes of renal bleeding [10, 11]. Congenital or acquired arterial injuries include active bleeding, aneurysms, pseudoaneurysms, arteriovenous fistulas (AVFs), and arteriovenous malformations (AVMs).

While parenchymal lesions can resolve spontaneously, arterial injuries may require immediate treatment due to severe bleeding and life-threatening hemodynamic instability. TAE is the first-line option to treat penetrating or iatrogenic trauma when conservative treatment has failed, or in cases of bleeding angiomyolipoma [12]. Minimally invasive approaches, such as transarterial embolization (TAE) are preferred over surgery due to rapid recovery periods and shorter hospital stays, which allows for preservation of renal tissue in severe cases [13, 14]. However, the requirement for contrast medium injections [15] and the potential risk of necrosis of healthy renal tissue may lead to a decline in renal function over time. The development of microcatheters and embolic agents expands the spectrum of treatment for patients and lesion types, allowing for proximal and/or distal occlusion. In the literature, there is a lack of evaluation of clinical outcomes of emergency TAE for renal injuries. Retrospective studies are either old, involve a small number of patients [16], or include TAE in a particular tumoral or iatrogenic context [17, 18].

The aim of this retrospective single-center study was to evaluate the clinical and functional outcomes of emergency renal TAE for renal injury.

## Methods

### Study population

Patients treated by TAE in emergency setting for renal injuries in our institution between January 1st, 2013 and January 1st, 2024 were retrospectively reviewed. The inclusion criteria were all patients ≥ 18-years-old treated by emergency TAE for renal vascular injuries of (1) traumatic, (2) iatrogenic, (3) tumoral origins or (4) following ruptures of arterial malformation (AVFs and AVMs). The exclusion criteria were: (1) scheduled TAE of angiomyolipoma, (2) preoperative tumoral embolizations, (3) scheduled TAE of complete renal parenchyma, (4) insufficient/incomplete clinical information, and (5) patients lost to follow-up within 30 days.

### Clinical data

Patient demographic data included age and gender. Medical data included comorbid conditions and anticoagulant therapy (anticoagulant and antiplatelet therapy) before TAE. Data were collected from electronic medical records. Biological data included haemoglobin levels just before TAE. Transfusion requirements included the number of red blood cell (RBC) and plasma units transfused between the 24 h before and the day of the procedure. The bleeding aetiology was based on the clinical history and preoperative CT scan data. Hemodynamic instability was defined as a decrease in blood pressure requiring the use of amines.

### Pre-prcoedural imaging

All patients underwent an abdominal CT scan (SOMATOM DEFINITION AS 64, Siemens AG, Medical Solution, Erlangen, Germany) prior to TAE. Unenhanced and contrast-enhanced abdominal CT at the arterial (35 s) and portal phases (80 s) were performed as standard of care in our institute. All preoperative CT images were analyzed by a radiologist with 10 years of experience in emergency imaging. The presence of perirenal and/or hemoretroperitoneum was assessed. Active bleeding was considered when iodine extravasation appeared at the arterial phase and increased at portal phase. A pseudoaneurysm was defined as hyperattenuating (contrast-enhanced) smooth-walled sac adjacent to an artery, with a communication [19]. For blunt traumas, a grading was conducted using the American Association for the Surgery of Trauma (AAST) score [20, 21].

### Procedural data

All TAEs were performed by 1 of the 7 different interventional radiologists, following a multidisciplinary decision between surgeons, clinicians, and radiologists. Patients with suspected AVM or AVF were transferred to the operating room. The transfer of patients with a pseudoaneurysm and/or active bleeding to the operating room depended on several criteria: the etiology, the patient’s condition (age, underlying heart disease), hemodynamic parameters (blood pressure, heart rate), the need for rapid resumption of anticoagulant therapy, and findings on preoperative imaging (presence and extent of hemoretroperitoneum). The right common femoral artery was accessed routinely. Selective catheterism of right/left renal artery was conducted using a 4F SHK or Cobra catheter. One patient treated with vascular plug required 6F catheter We obtained angiographic subtraction images with contrast iodine media (15 ml at 3 ml/s) to confirm arterial injuries and determine the injury type, which included arteriovenous fistula, arteriovenous malformation, pseudoaneurysm, aneurysm, and/or active bleeding. Supraselective catheterization was systematically performed using a 2.7F Progreat® microcatheter (Terumo, Tokyo, Japan), except if occlusion with a plug was decided. TAEs were performed using microcoils (IDC® and Interlock®, Boston Scientifics), microparticles (Embospheres®, Meritt Medical), Amplatzer® Vascular Plugs (Abbott medical), N-butyl-2-cyanoacrylate (Glubran2®, GEM, Viareggio, Italy), gelatine sponges (Gelitaspon®, Gelitamedical, GmbH, Eberbach, Germany), or a combination of several embolic agents. The choice of combination of embolic agents, was used in case of multiple target arteries. After occlusion of target arteries, angiograms were systematically performed to confirm the occlusion of renal injuries. After the procedures, introducers were left in place, and patients were monitored in either medical or intensive care units. The introducer was systematically removed 24 h after the end of the procedure, provided there was no continued drop in hemoglobin levels. The diameter of target arteries was measured based on angiographic data. The procedure duration was calculated from the first image to the last fluoroscopy image. Arterial injury was defined as proximal if it originated from the renal artery or segmental arteries, intermediate if it involved the interlobar arteries, and distal if it was located in the interlobular arteries.

### Patient follow up

Patient outcomes, including complications related to the procedure, need for repeated TAE, and death, were extracted from patient records.

Technical success was defined as the absence of vascular lesion opacification and/or cessation of bleeding at the end of the procedure. Clinical success was defined as the resolution of bleeding signs without the need for repeat TAE, surgery, death related to massive blood loss during this period, without functional impairment (> 50% of parenchyma volume or onset of chronic kidney disease) following TAE. Post-operative infection was defined as an inflammatory syndrome associated with an inflammatory enhancement at the periphery of the haematoma on the follow-up CT scan in the days following the procedure. Serum creatinine was assessed just before and 15–30 days after the procedure. Complications were graded using the SIR classification [22]. Overall survival was defined as the number of days from the procedure to the latest available information or death.

### Statistical analysis

Results are expressed as frequencies and percentages for categorical variables and as medians and interquartile ranges for continuous variables. Categorical variables were evaluated using the Chi-square test, while continuous variables were assessed using the Student t-test. Odds ratios (OR) and 95% confidence intervals were calculated. Statistical significance was defined as *p* < 0.05. All statistical analyses were conducted using GraphPad software (version 8.4.2).

### Ethical considerations

This study was approved by our institute’s Ethical Committee (IRBN112021).

## Results

### Patient population

**Fig. 1:**
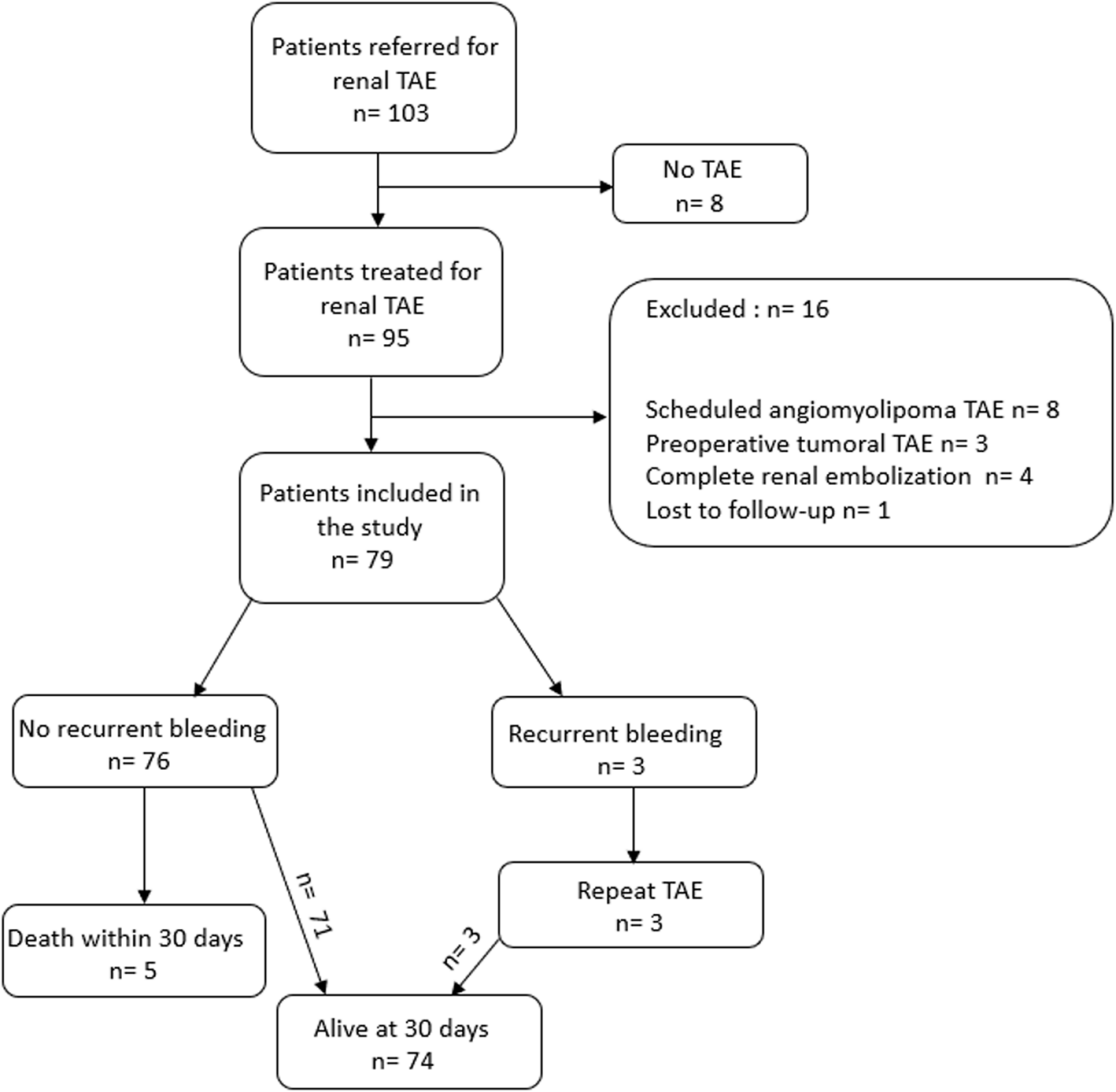
Study population flow chart

Between January 1st, 2013 and January 1st, 2024, 103 (47(59.5%)) males patients were referred to our department for renal TAE. Eight patients with active bleeding observed on preoperative CT scan were not treated due to the disappearance of active bleeding on procedural angiogram. In total, 79 patients were thus included. Detailed patient characteristics are presented in Table 1. The flow chart of the patient sample population is shown in Fig. 1. The median age was 60[39–73] years. The median hemoglobin level was 8.9[7.6–11] g/dl. RBC transfusions were necessary for 37(46.8%) patients, and plasma transfusions for 14(17.7%) patients. Hemodynamic instability was detected in 29(25.3%) patients. All anticoagulant therapy was stopped until the technical success of the procedure was achieved. The aetiology of the vascular injuries is detailed in Table 2.

**Table 1:** Demographic, medical and biological data of the study population (*N* = 79)

Variables	Total *N* = 79
Age, y^a^	60[39–73]
Male, n (%)	47(59.5)
Comorbidities, n (%)	
Diabetes	11(13.9)
Coronaropathy	20(25.3)
HBP	31(39.2)
Dyslipidemia	22(27.8)
CKD	20(25.3)
Active cancer	9(11.4)
Anticoagulation therapy, n (%)	11(13.9)
Antiaggregant therapy, n (%)	4(5.1)
Hemodynamic instability, n (%)	20(25.3)
Biology	
Hb (mg/dl)^a^	8.9[7.6–11]
TP^a^	77[68–90.5]
TP < 70%, n (%)	18(22.8)
INR^a^	1.14[1.04–1.27]
INR > 1.5, n (%)	5(6.3)
Pre-operative CT, n (%)	
Aneurysm / Pseudoaneurysm	38(48.1)
Active bleeding	46(58.2)
Hemoretroperitoneum	44(55.7)
Bleeding on renal graft	6(7.6)
RBC transfusion, n (%)	37(46.8)
RBC units / patient^a^	2[2–4]
Plasma unit transfusion, n (%)	14(17.7)
Plasma units / patient^a^	2[2–2]

*CKD* Chronic Kidney Disease, *HBP* High Blood Pressure, *Hb* Hemoglobin, *INR* International Normalized Ratio, *RBC* Red Blood Cell

^a^Values expressed in median [1IQR-3IQR]

**Table 2:** Bleeding aetiology of the study population (*N* = 79)

Variables	Total *N* = 79	Hemodynamic instability *N* = 20	Hemodynamic stability *N* = 59	*p-value*
Trauma, n (%)				
Blunt trauma	**21(26.6)**	**6(30.0)**	**15(25.4)**	**0.35**
AAST III	7(8.9)	2(10.0)	5(8.5)	
AAST IV	5(6.3)	2(10.0)	3(5.1)	
AAST V	7(8.9)	2(10.0)	5(8.5)	
Penetrating trauma	2(2.5)	0(0)	2(3.4)	
Iatrogenic origin, n (%)	**34(43.0)**	**4(20.0)**	**30(50.8)**	**< 0.001**
JJ stent, n (%)	2(2.5)	0(0)	2(3.4)	
Percutaneous Renal Biopsy, n (%)	17(21.5)	1(5.0)	16(27.1)	
Catheterization, n (%)	3(3.8)	2(10.0)	1(1.7)	
Partial Nephrectomy, n (%)	12(15.2)	1(5.0)	11(18.6)	
Tumoral bleeding, n (%)	**12(15.2)**	**4(20.0)**	**8(13.6)**	**0.25**
Clear-cell carcinoma	1(1.3)	1(5.0)	0(0)	
Cyst	3(3.8)	1(5.0)	2(3.4)	
Angiomyolipoma	8(10.1)	2(10.0)	6(10.2)	
Other causes, n (%)	**12(15.2)**	**6(30.0)**	**6(10.2)**	**0.05**
Rupture of renal aneurysm, n (%)	2(2.5)	2(10.0)	0(0)	
Extrinsic compression of renal aneurysm, n (%)	1(1.3)	0(0)	1(1.7)	
Rupture of arterio-venous malformation, n (%)	1(1.3)	0(0)	1(1.7)	
Spontaneous bleeding, n (%)	7(8.9)	4(20.0)	3(5.1)	
Infection, n (%)	1(1.3)	0(0)	1(1.7)	

*AAST* American Association for the Surgery of Trauma

### Procedure

**Fig. 2:**
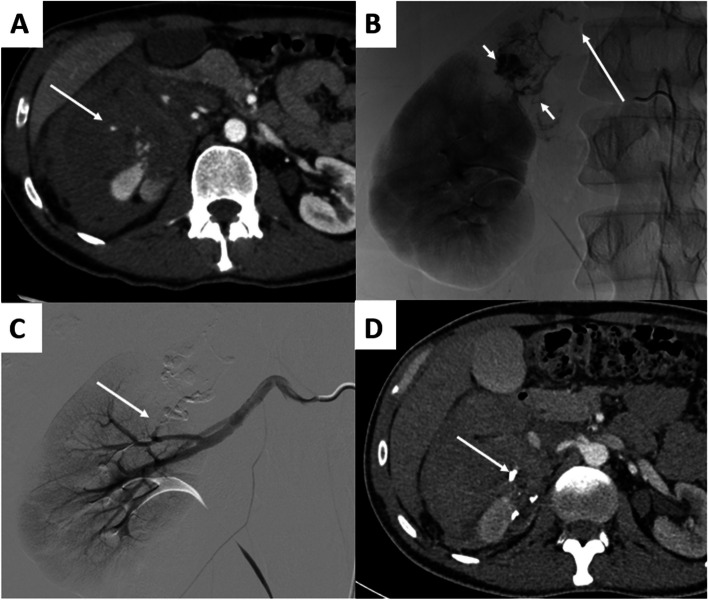
A 45-year-old patient presented with spontaneous right lumbar pain in the context of a known angiomyolipoma. **A** abdominal CT scan in axial section at arterial phase showed active bleeding with a large retroperitoneal hematoma (arrow). The tumor lesion was not distinctly visualized. **B** Angiography revealed tumor hypervascularization (short arrows) and active bleeding (long arrow). **C** After embolization with NBCA/lipiodol mixture (ratio 1/1), the angiography showed no active bleeding and no tumor opacification (arrow). **D** The CT scan performed 5 days later shows a reduction in the size of the retroperitoneal hematoma and lipiodol deposit (arrow)

**Fig. 3:**
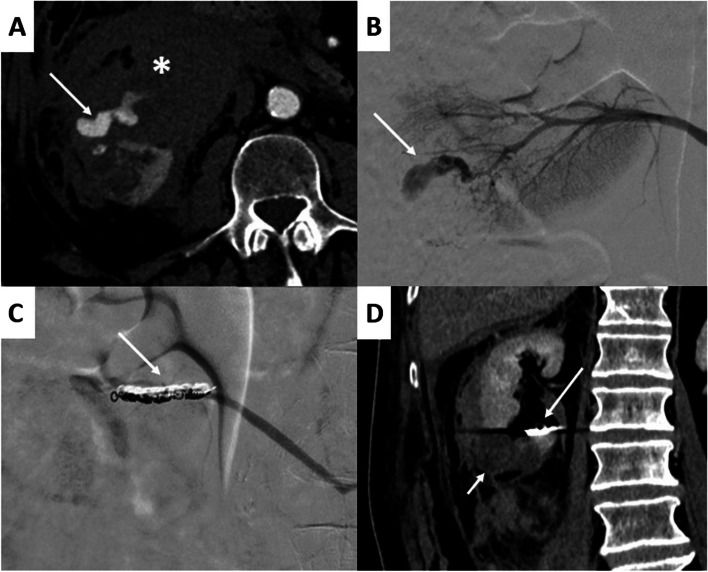
A 63-year-old man who underwent a renal biopsy 4 h earlier. The patient presented with a sudden drop in hemoglobin levels accompanied by pallor. **A** The axial view of an arterial-phase contrast-enhanced abdominal CT scan showed a large right retroperitoneal hematoma (*) along with active renal bleeding (arrow) from the lower pole of the right kidney. **B** Angiography of the right lower polar artery confirmed active bleeding (arrow). **C** Follow-up angiography after placement of two microcoils (arrow) shows cessation of the active bleeding. **D** Contrast-enhanced CT scan at portal phase in coronal section revealed limited necrosis of the lower pole (short arrow) with coil artifacts (long arrow)

The per-procedure details are presented in Table 3. Thirty-six (45.6%) patients had ≥ 1 pseudoaneurysm, 3 patients had AVF and 1 patient had AVM. In patients with pseudoaneurysms, 26 patients had 1 pseudoaneurysm, 7 patients had 3 pseudoaneurysms, 2 patients had 3 pseudoaneurysms and 1 patient had 4 pseudoaneurysms. Procedures using coils alone occurred in 39(49.3%) procedures, NBCA was used in 24(30.4%) procedures, and microparticles were used in 4(5.1%) procedures (Figs. 2 and 3). The median diameter of embolized arteries was 2.4[1.8–3.0] mm. After the procedure, 64/79(84.8%) benefited from control CT scan. The remaining 12 patients were either early deceased (*n* = 3), young (*n* = 2), or patients with hemodynamic stability that did not required a follow-up CT scan (*n* = 7).

**Table 3:** Procedural data of the study population (*N* = 79)

Variables	Total *N* = 79
Angiographic findings, n (%)	
≥ 1 pseudoaneurysm, n (%)	36(45.6)
Number of pseudoaneurysms / patient, n (%)	
1	26(32.9)
2	7(8.9)
3	2(2.5)
4	1(1.3)
Aneurysm, n (%)	2(2.5)
AVF, n (%)	3(3.8)
Arteriovenous malformation, n (%)	1(1.3)
Active bleeding only	37(46.8)
Localization of arterial injury, n (%)	
Proximal	12(15.2)
Intermediate	34(43.0)
Distal	33(41.8)
Embolic Agents, n (%)	
Coils alone	39(49.3)
NBCA	24(30.4)
Microparticles	4(5.1)
Plug	3(3.8)
Stenting	1(1.3)
NBCA + coils	2(2.5)
Coils + Gelatine sponge	3(3.8)
Coils + microparticles	2(2.5)
Gelatine sponge	1(1.3)
Number coils / patient, n (%)	2[2–3]
Size of target artery (mm)^a^	2.4[1.8–3]
Size of pseudoaneurysm (mm)^a^	10[7–18]
Duration of procedure (min)^a^	60[39–73]

*AVF* Arteriovenous Fistula, *NBCA* N-Butyl-Cyanoacrylate

^a^Values expressed in median[1IQR-3IQR]

### Complications

**Fig. 4:**
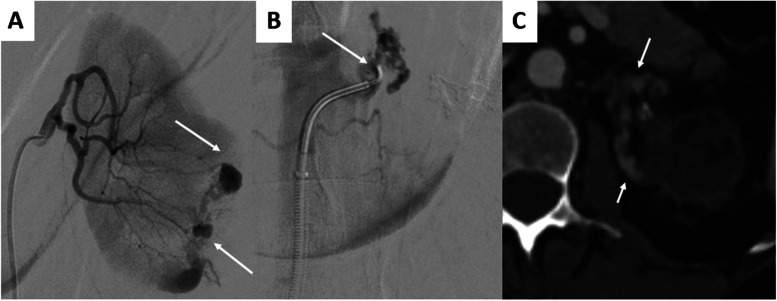
A 76-year-old patient presented with left lumbar pain and was found to have a large angiomyolipoma with active bleeding. **A** Angiography revealed two distinct bleeding sites from two different branches of the left renal artery (arrows). **B** After embolization of the lower branch with NBCA/Lipiodol (ratio 1/1), an attempt to catheterize the upper branch was performed, but an arterial dissection of renal arterial appeared (arrow). **C** The CT scan in axial section at arterial phase performed two years later shows atrophy of the upper pole of the left kidney (arrows)

There were no severe adverse events. Three patients had mild (grade I) adverse events and five patients had moderate (grade II) adverse events. Four patients had postoperative pain within 4 h following the procedure, requiring morphine infusions. One patient had infection of hematoma, requiring antibiotic therapy. One patient had a hematoma at the puncture site treated by compression, with a favorable progression. There was one case of renal artery dissection due to catheterization in a 76-year-old patient, followed by renal atrophy (Fig. 4). A 60-year-old patient treated with microparticles showed signs of diffuse cortical infarcts due to non-target embolization, following by left renal atrophy.

### Outcomes

Technical success was achieved in all patients. Clinical success was reported among 74(93.7%) patients. Detailed clinical outcomes after TAE are presented in Table 4. After a median follow-up time of 7[1.5–35.5] months, 9(11.4%) patients died, including 5(6.3%) patients who died within 30 days following the procedure. Two female patients, aged 20 and 39, died of irreversible cerebral oedema following multiple traumas. A 75-year-old patient died of acute respiratory distress syndrome with acute renal failure in the context of multiple myelomas. An 84-year-old female died following a spontaneous kidney hematoma under anticoagulant therapy. An 87-year-old female died 3 days after the perforation of a renal artery during a transcatheter aortic valve implantation procedure, despite successful covered stent placement. Three (3.8%) patients treated with coils alone had a repeated TAE due to persistence of pseudoaneurysm without active bleeding on control CT scan, and were successfully embolized with coils (*n* = 2) or NBCA (*n* = 1). The median creatinine level was 115[89–169.5] mmol/l before the procedure and after the procedure 95[80.25–167.25] (*p* > 0.05).

**Table 4:** Complications and outcomes of the study population (*N* = 79)

Variables	Total *N* = 79
Technical Success, n (%)	79(100)
Clinical success, n (%)	74(93.7)
Mortality during follow-up, n (%)	9(11.4)
30-day mortality, n (%)	5(6.3)
Creatinine level before procedure (mmol/l)^a^	115[89–169.5]
Creatinine level after procedure (mmol/l)^a^	95[80.25–167.25]
Per-operative Complications, n (%)	8(10.1)
Non-target embolization	1(1.3)
Haematoma at puncture site	1(1.3)
Pain requiring morphine therapy	4(5.1)
Ischemia	1(1.3)
Postoperative infection	1(1.3)
SIR grading complication, n (%)	
I	3(3.8)
II	5(6.3)
III	0(0)
IV	0(0)
V	0(0)
Repeated TAE, n (%)	3(3.8)
Salvage surgery, n (%)	0
Duration of follow-up (months)^a^	7[1.5–35.5]

*TAE* Transarterial embolization, *SIR* Society of Interventional Radiology

^a^Values expressed in median[1IQR-3IQR]

## Discussion

This retrospective monocentric study reports excellent outcomes following emergency TAE of arterial kidney injury, without requirement for salvage surgery, a low rate (3.8%) of repeated TAE, and no severe adverse events. This is in line with another study including 86 procedures performed on 76 patients which showed a high rate of clinical success, with only 1 salvage surgery [23].

The choice of renal hemostasis embolization should consider initial clinical presentation, aetiology, patient comorbidities, use of anticoagulant therapy, and presence of underlying arterial lesions. TAE allows for the cessation of bleeding with a low technical failure rate but carries the risk of renal function deterioration due to necrosis of functional nephrons. Moreover, active renal bleeding may spontaneously cease due to the natural compression of the perirenal or retroperitoneal hematoma. It is noteworthy that patients with hemodynamic stability more frequently presented with an iatrogenic origin of bleeding (*p* < 0.001). This can be attributed to several factors. First, these patients are often already hospitalized at the time of the bleeding episode, allowing for rapid imaging and prompt management. Consequently, the decrease in hemoglobin levels tends to be moderate. Moreover, an iatrogenic etiology may prompt clinicians to adopt a more proactive therapeutic approach, aiming to minimize both clinical complications and psychological distress. While no randomized studies have directly compared active surveillance with transarterial embolization (TAE) in hemodynamically stable patients, it is important to recognize that the purpose of embolization extends beyond merely stopping the bleeding. Psychological repercussions of the bleeding event, the logistical challenges of closely monitoring hemoglobin levels during conservative management, and the necessity of resuming anticoagulant therapy are all factors that strongly support the decision to perform embolization, even in the absence of hemodynamic instability.

However, the mortality rate in the absence of active treatment is not known. The absence of salvage surgery in this study demonstrates the clinical success of TAE, even among patients with initial hemodynamic instability. The mortality rate is low in our sample, and is lower than those demonstrated in cases of digestive embolizations [24] or spontaneous hematomas [25, 26]. This can be explained by a low rate of hemodynamic instability in our study, a higher hemoglobin level, a lower transfusion rate, and a younger age compared to studies reporting outcomes of gastrointestinal bleeding [24]. In our study, deaths that occurred within 30 days included (1) patients whose renal blunt trauma was associated with cranial trauma leading to cerebral oedema and irreversible cerebral hypertension, and (2) patients with factors worsening their clinical prognosis, such as those who were elderly, frail, or already hospitalized and whose deaths were not directly attributable to renal bleeding.

Our study reports a wide range of embolic agents used to treat arterial injury, contrary to the previous study of Öcal and al, in which coils alone were used in 68/86(79.1%) procedures [23]. Renal vascularization is terminal, which prevents the risk of recurrence via anastomoses. Coils were the most commonly used embolization agent, either alone or in combination, in line with literature [27]. This embolic agent is quick and requires little experience. However, arterial occlusion may necessitate multiple coils, with delayed thrombus formation in cases of coagulopathy [28]. Interestingly, three patients who needed repeated TAE were initially treated with coils alone. It is worth noting that two patients were treated with plugs to address post-biopsy AVF and spontaneous AVM rupture, without recurrence. The use of plugs offers the advantage of achieving proximal, immediate, and rapid arterial occlusion [29, 30]. This may reduce procedural duration and fluoroscopy time, especially when treating large arteries. In one patient, the placement of a 10 mm plug required the use of a 6F catheter. Additionally, NBCA can occlude small-caliber arteries < 1.5 mm, where the use of microcoils is challenging. However, its application can be tricky. The injection of the NBCA/lipiodol mixture should be slow to prevent the risk of reflux along the microcatheter and non-target embolization.

The functional outcomes of our study are particularly good. None of the patients required dialysis following TAE and creatinine level did not significantly decrease after TAE. It is interesting to note that the diameter of embolized arteries was very small in our study, and was notably smaller than what has been reported in retrospective studies on digestive embolizations [31]. Distal occlusion of target artery was possible due to current use of microcatheters, except for 3 procedures in which plugs alone were used. Nevertheless, two patients had renal atrophy following TAE, due to arterial dissection with Cobra catheter, and non-target embolization following utilisation of microparticles. The use of microparticles must be approached with caution [32]. The end point of embolization with microparticles is difficult to identify. Moreover, use of microparticles is contraindicated in cases of suspected AVF or AVM, due to the risk of systemic migration. We do not recommend its use for treating post-biopsy bleeding, given the frequency of associated AVF or AVM [33].

Our study has some limitations. First, this is a retrospective, single-center study, with inherent biases associated with such studies. We did not compare TAE to active surveillance, which may be a viable therapeutic approach in stable patients. Moreover the sample size of the study population was relatively small. The evolution of renal function must be interpreted with caution. Decreases in renal function can be caused by a variety of factors including contrast-induced nephropathy, haemorrhagic shock and dehydration, especially in those with pre-existing chronic kidney disease. Moreover, patients can maintain their renal function even after a renal ischemic complication, with contralateral renal hypertrophy. This allowed us to classify as clinical failure the two patients who experienced renal atrophy after TAE due to renal artery dissection and non-target embolization with microparticles. It is worth noting that these two patients preserved their renal function in the months following the embolization.

In conclusion, this study demonstrates that emergency renal TAE is associated with a high rate of clinical success and avoids the need for nephrectomy, even in patients with hemodynamic instability, without severe adverse events.

## Data Availability

The datasets used and analyzed during the current study are available from the corresponding author on request.

## Abbreviations

AVF: Arteriovenous fistula
AVM: Arteriovenous malformation
AAST: American Association for the Surgery of Trauma
TAE: Transarterial embolization
NBCA: N-Butyl Cyanoacrylate
SIR: Society of Interventional Radiology
VAPA: Visceral pseudoaneurysm
